# Supplementary material for: Exploring patterns enriched in a dataset with contrastive principal component analysis
Source: Nat Commun. 2018 May 30;9:2134. doi: 10.1038/s41467-018-04608-8 (PMC5976774; doi:10.1038/s41467-018-04608-8)
Supplement: Supplementary file 1 — Supplementary Information [file 41467_2018_4608_MOESM1_ESM.pdf]

Exploring Patterns Enriched in a Dataset with Contrastive Principal Component Analysis

Abid et al.

Supplementary Information

Supplementary Figures

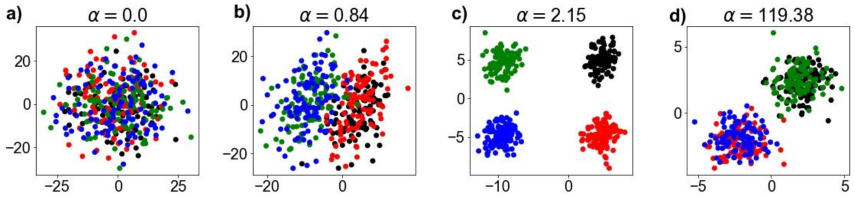

Supplementary Figure 1: **cPCA discovers different subgroup structures with different values of α in a synthetic dataset.** The data has dimensionality 30. The target data contains four subgroups, **red**, **blue**, **green**, **black**, each with 100 points. For each of the first 10 features, all points are sampled from $\mathcal{N}(0, 10)$; for each of the second 10 features, **green**/**blue** are sampled from $\mathcal{N}(3, 1)$ and **red**/**black** from $\mathcal{N}(-1.5, 1)$; for each of the last 10 features, **green**/**black** are sampled from $\mathcal{N}(-1.5, 1)$ and **red**/**blue** from $\mathcal{N}(1.5, 1)$. The background data contains 400 points sampled from the same distribution; each of the first 10 features from $\mathcal{N}(0, 10)$, the second 10 from $\mathcal{N}(0, 3)$, and the third 10 from $\mathcal{N}(0, 1)$. After this, a random rotation is applied to the both datasets to make it non-trivial to find the directions that separate the subgroups in the target dataset. We remark that the purpose of the synthetic dataset is to demonstrate the behavior of cPCA; it is not meant to capture the complexity found in the structure of biological (e.g. genomic) datasets. The results of cPCA with different values of α are shown above, where $\alpha = 0$ corresponds to PCA. (a) PCA is unable to resolve the subgroups since the variance along the the last 10 dimensions is significantly larger than the rest. (b) With a small α, the last 10 dimensions are removed by the background data. cPCA selects cPCs from the first 10 dimensions that have larger target variance than the second 10, allowing us to discriminate between **green**/**blue** and **red**/**black**. (c) With an intermediate value of α, cPCA selects cPCs among the first 20 features, allowing us to separate all four subgroups. (d) with a very large α, cPCA will only select the second 10 features that have the smallest background variance, allowing us to discriminate between **red**/**blue** and **green**/**black**.

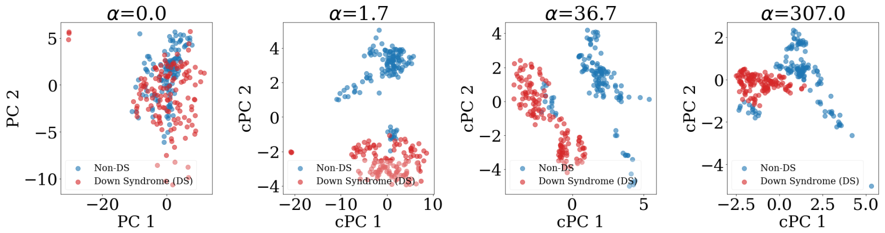

(a)

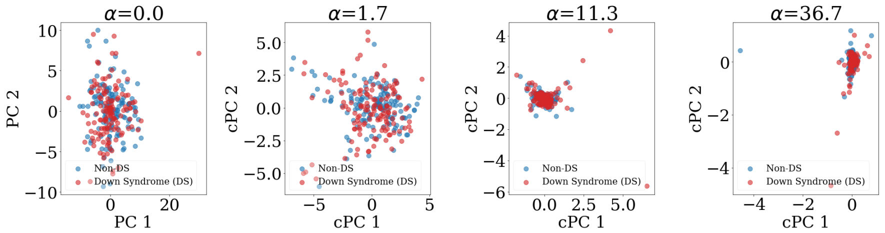

(b)

Supplementary Figure 2: **Mice Protein Expression Dataset** (a) Here, we show the full results of applying cPCA to the mice protein expression dataset. (b) How likely is it that cPCA is discovering these clusters by chance? We can get an idea by shuffling the foreground and background data, as well as the labels for the foreground data, and running cPCA again. A representative simulation is shown here. With this random shuffling, the subgroup structure no longer shows up. This indicates that it is unlikely that cPCA discovered the clusters in (a) by chance.

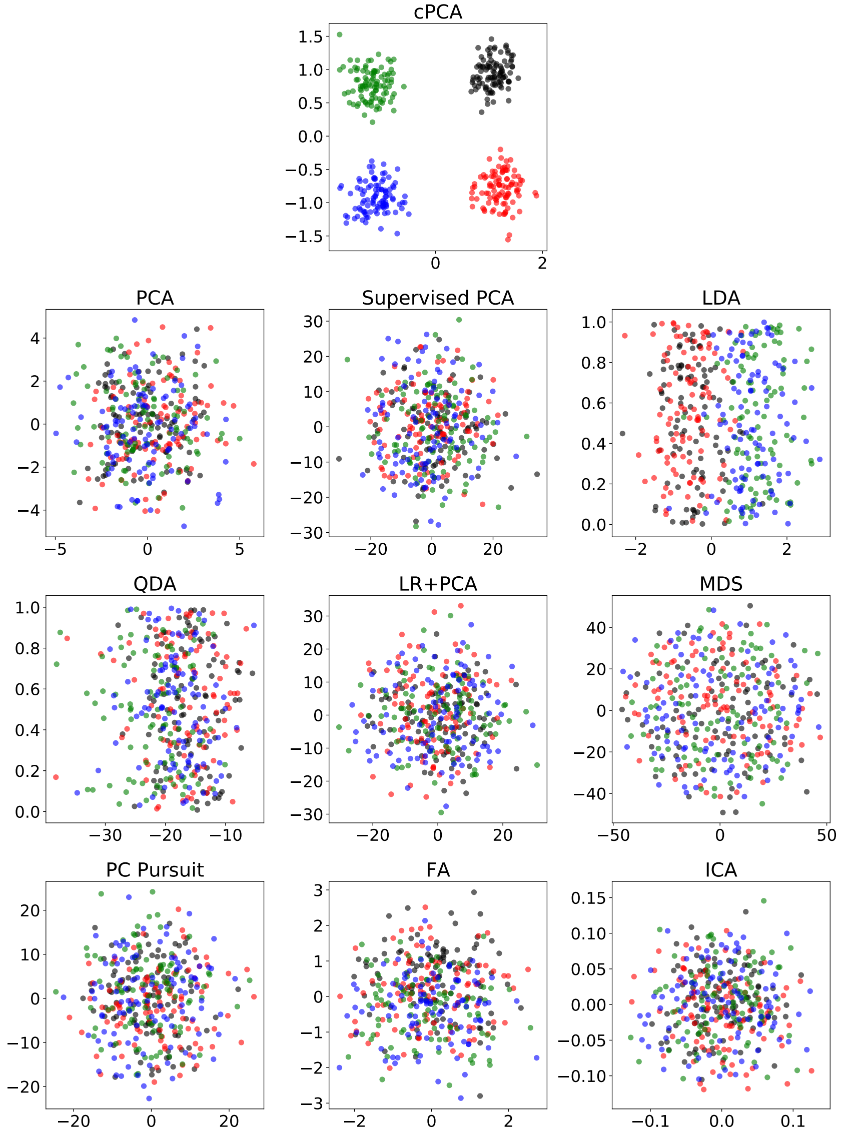

Supplementary Figure 3: **A comparison of** 10 **dimensionality-reduction techniques on the synthetic data in Supplementary Figure 1**. The result shows that cPCA is the most effective technique to discover and separate subgroups within the data. The methods for comparison are PCA, supervised PCA[1], linear discriminate analysis (LDA)[2], quadratic discriminate analysis (QDA)[3], LR+PCA (PCA performed on most useful features for linear regression) , multidimensional scaling (MDS)[4], principal component pursuit (PCP)[5], factor analysis (FA)[6], and independent component analysis (ICA)[7]. All methods except PCP uses the implementation in *sklearn*[8]. PCP uses the implementation in its own paper[5].

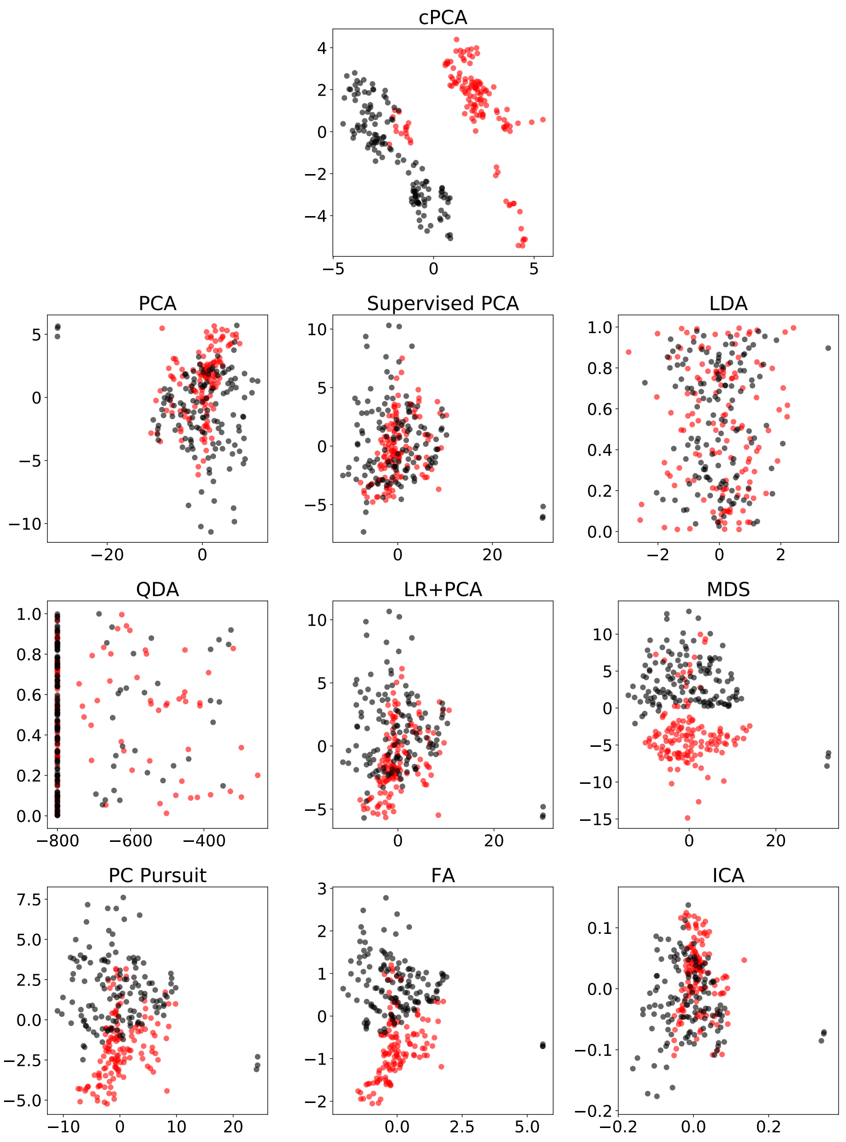

Supplementary Figure 4: **A comparison of** 10 **dimensionality-reduction techniques on the Mice protein data in Supplementary Figure 2.** The result shows that cPCA is the most effective technique to discover and separate subgroups within the data. The methods are the same as those used in Supplementary Figure 3.

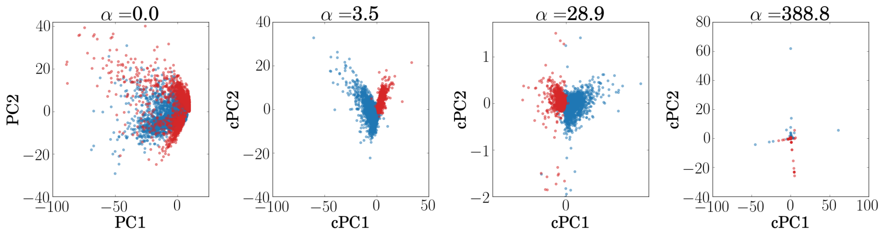

(a)

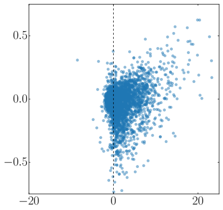

(b)

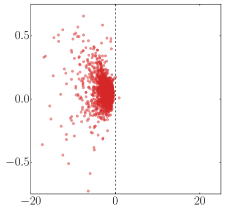

(c)

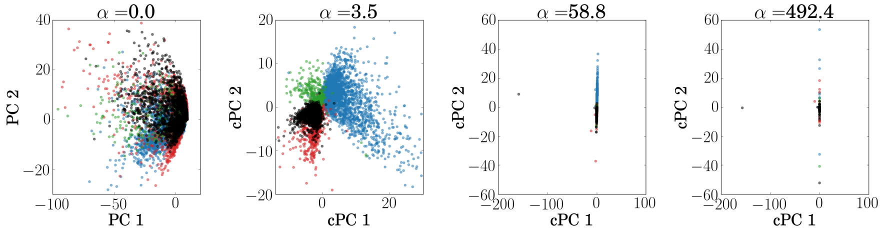

(d)

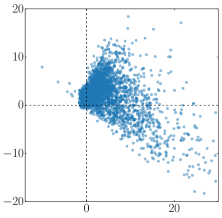

(e)

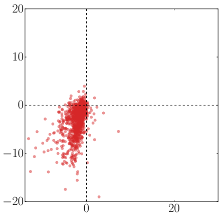

(f)

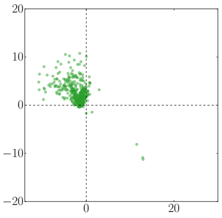

(g)

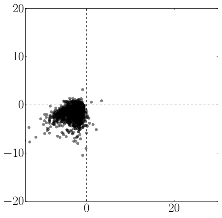

(h)

Supplementary Figure 5: **More results on the Single Cell RNA-Seq Dataset.** (a) Results on a dataset consisting of a mixture of 2 cell samples. (b,c) Each cell sample is plotted separately for the scatterplot corresponding to the third panel ($\alpha = 28.9$) in (a). (d) Results on a dataset consisting of a mixture of 4 cell samples. (e-h): Each cell sample is plotted separately for the second panel ($\alpha = 3.5$) in (d).

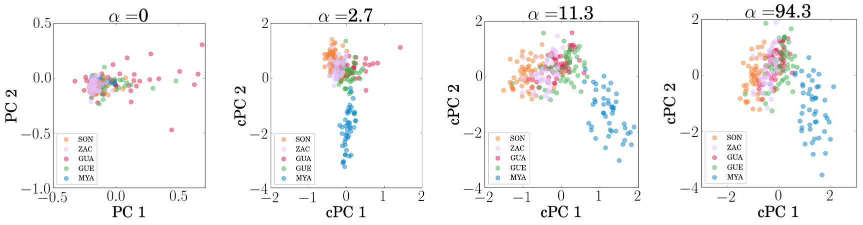

(a) (b) (c) (d)

Supplementary Figure 6: Results of cPCA on the Mexican Ancestry Dataset with different values of α.

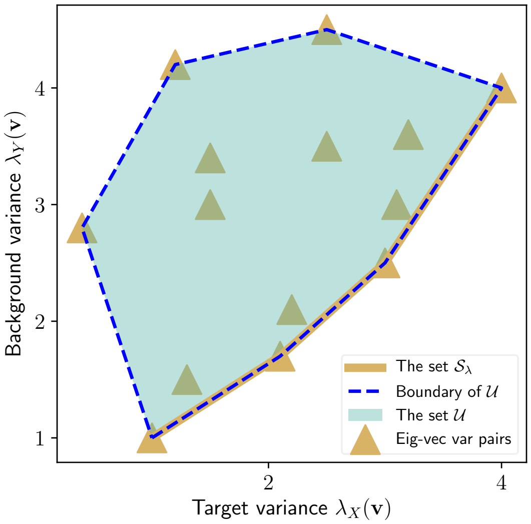

Supplementary Figure 7: The geometry in a simultaneously diagonalizable system in Supplementary Example 1 The set of target-background variance pairs $\mathcal{U}$ is plotted as the teal region for some randomly generated target C_X and background data C_Y. It consists the convex hull of the brown triangles, which are variance pairs for the common eigenvectors of C_X and C_Y. The set of most contrastive directions $\mathcal{S}_\lambda$ is the lower-right brown line, and the boundary of $\mathcal{U}$ is the blue dashed line.

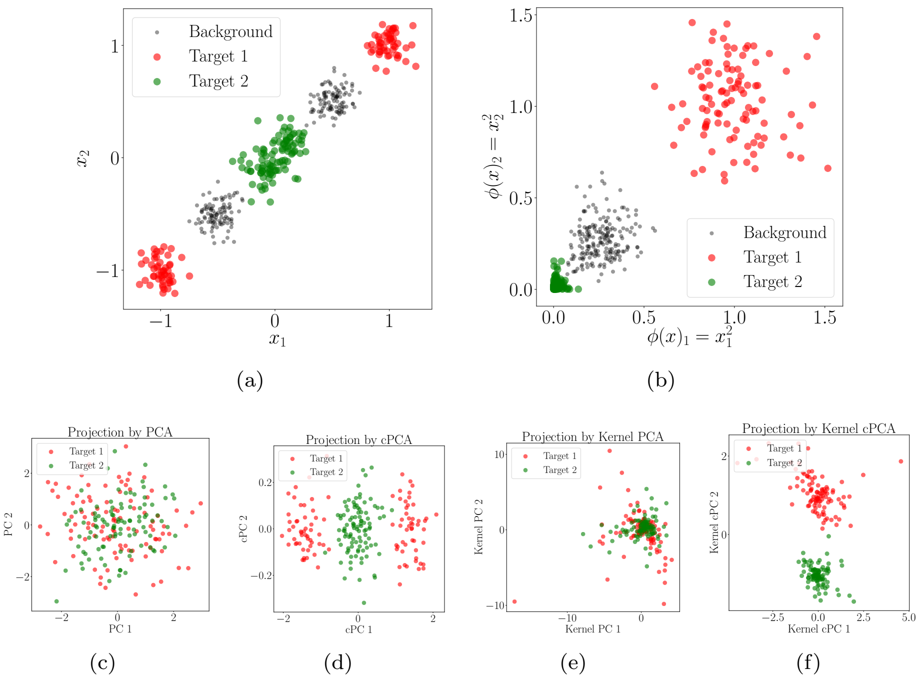

Supplementary Figure 8: **A simulation example for kernel cPCA in Supplementary Example 2.** (a) the data on the first two dimension x_1, x_2, and the two subgroups in the target data (red/ green) are not linearly separable. (b) the data on two non-linear features $\phi(x_1) = x_1^2, \phi(x_2) = x_2^2$, where the two subgroups become linearly separable. (c-f) The results by (c) PCA, (d) cPCA, (e) kernel PCA, (f) kernel cPCA.

Supplementary Methods

Algorithm 1 cPCA for a Given α

Inputs: target data $\{\mathbf{x}_i\}_{i=1}^n$; background data $\{\mathbf{y}_i\}_{i=1}^m$; contrast parameter α; the number of components k.

Centering the data $\{\mathbf{x}_i\}_{i=1}^n$, $\{\mathbf{y}_i\}_{i=1}^m$.
Calculate the empirical covariance matrices:

$$C_X = \frac{1}{n}\sum_{i=1}^{n} \mathbf{x}_i \mathbf{x}_i^T, C_Y = \frac{1}{m}\sum_{i=1}^{m} \mathbf{y}_i \mathbf{y}_i^T.$$

Perform eigenvalue decomposition on

$$C = (C_X - \alpha C_Y).$$

Compute the the subspace $V \in \mathbb{R}^k$ spanned by the top k eigenvectors of C.
Return: the subspace V.

Algorithm 2 cPCA with Auto Selection of α

Inputs: target data $\{\mathbf{x}_i\}_{i=1}^n$; background data $\{\mathbf{y}_i\}_{i=1}^m$; list of possible contrastive parameters $\{\alpha_i\}$; the number of components k; the number of α's to present p.

for each α_i **do**
  Compute the subspace V_i using Algorithm 1 with the contrast parameter set to α_i.
end for
for each pair V_i, V_j **do**
  Compute the principal angles $\theta_1 \dots \theta_k$ between V_i, V_j
  Define the affinity $d(V_i, V_j) = \prod_{h=1}^k \cos\theta_h$
end for
With $D_{ij} = d(V_i, V_j)$ as an affinity matrix between subspaces, do spectral clustering on D to produce p clusters.
for each cluster of subspaces $\{c_i\}_{i=1}^p$ **do**
  Compute its medoid, V_i^* the subspace defined as

$$V_i^* \stackrel{\text{def}}{=} \arg\min V \in c_i \sum_{V' \in c_i} d(V, V')$$

  Let α_i^* be the contrast parameter corresponding to V_i^*.
end for
Return: $\alpha_1^* \cdots \alpha_p^*$ and the subspaces $V_1^* \cdots V_p^*$.

Supplementary Notes

1 Supplementary Note 1: More Empirical Results

1.1 Different Alphas Reveal Different Subgroups

In Supplementary Figure 1, we present an example with synthetic data. In this example, cPCA discovers different subgroup structures with different values of α. See the figure caption for more details.

1.2 Data-Dependent Standardization

Because the PCA calculates the directions in a dataset with the highest covariance, it is highly sensitive to the units used to measure each feature. As a consequence, when different units are used to measure different features, it is common to standardize the data by dividing each column of the data matrix by its standard deviation, thereby ensuring that each feature has unit variance[9, 10]. However, this procedure has a drawback: noisy features with low variance are inflated to have the same variance as the most significant features; in fact, some sources suggest that standardization should not be used unless low-variance features are removed first[11].

As an alternative, cPCA can be used as a dimensionality-reduction technique directly, without standardization, in cases when a reference, signal-free dataset is available as a background. By searching for features that contrast between the target and background, cPCA automatically provides a data-dependent standardization by eliminating those features that are equally noisy in both the target and background. We illustrate this with an example.

MHealth Measurements. The MHealth public dataset[12] consists of measurements from a variety of sensors (e.g. accelerometers, EKG, and gyroscopes) when subjects perform a series of different activities. In this example, our target dataset consists of sensor readings from a subject who is, at times, jogging and, at times, performing squats – two very different activities. We may wonder whether the sensor data can be used to visually distinguish these two activities. In Supplementary Figure **??**a, we show the result of applying PCA on the unstandardized data: the two activities cannot be distinguished visually.

We then take as a background dataset sensor readings from the subject when the subject is lying still. We assume this to be a signal-free reference, because most sensor readings will reflect their baseline noise levels. By performing cPCA, we see the two activities resolve clearly into two separate subgroups, as shown in Supplementary Figure **??**b – with no standardization needed. For this experiment, a larger range of initial values of α was used (0.1-10^6).

2 Supplementary Note 2: Theoretical Analysis

2.1 cPCA returns most contrastive directions

For any direction $\mathbf{v} \in \mathbb{R}^d_{\text{unit}}$, its target-background variance pair $(\lambda_X(\mathbf{v}), \lambda_Y(\mathbf{v}))$ fully determines its significance for cPCA. Intuitively, we might say that for any two directions $\mathbf{v}_1, \mathbf{v}_2 \in \mathbb{R}^d_{\text{unit}}$, $\mathbf{v}_1$ is a better contrastive direction than $\mathbf{v}_2$ if it has a larger target variance and a smaller background variance. Let us formalize this notion:

Definition 1. *(Contrastiveness) For two directions $\mathbf{v}_1, \mathbf{v}_2 \in \mathbb{R}^d_{unit}$, $\mathbf{v}_1$ is more contrastive than $\mathbf{v}_2$ with respect to the target and the background covariance matrices C_X and C_Y, written as $\mathbf{v}_1 \succ \mathbf{v}_2$, if one of the following is true:*

$$\begin{aligned} &(1) \quad \lambda_X(\mathbf{v}_1) \geq \lambda_X(\mathbf{v}_2), \text{ and } \lambda_Y(\mathbf{v}_1) < \lambda_Y(\mathbf{v}_2) \\ &(2) \quad \lambda_X(\mathbf{v}_1) > \lambda_X(\mathbf{v}_2), \text{ and } \lambda_Y(\mathbf{v}_1) \leq \lambda_Y(\mathbf{v}_2). \end{aligned}$$

We should note that the above definition provides a partial order for the directions in $\mathbb{R}^d_{\text{unit}}$. Then it is natural to say a direction $\mathbf{v}$ is most contrastive if there is no other direction more contrastive than $\mathbf{v}$. Formally

Definition 2. *Define the set of most contrastive directions $\mathcal{S}_{\mathbf{v}}$ and the corresponding set of target-background variance pairs $\mathcal{S}_\lambda$ to be:*

$$\begin{aligned} &\mathcal{S}_{\mathbf{v}} \stackrel{def}{=} \{\mathbf{v} \in \mathbb{R}^k_{unit} : \nexists\ \mathbf{v}' \in \mathbb{R}^d_{unit},\ s.t.\ \mathbf{v}' \succ \mathbf{v}\}, \\ &\mathcal{S}_\lambda \stackrel{def}{=} \{(\lambda_X(\mathbf{v}), \lambda_Y(\mathbf{v})) : \mathbf{v} \in \mathcal{S}_{\mathbf{v}}\}. \end{aligned}$$

It is also convenient to define $\mathcal{U}$ to be the set of target-background variance pairs for all directions in $\mathbb{R}^d_{\text{unit}}$, i.e. $\mathcal{U} \stackrel{\text{def}}{=} \{(\lambda_X(\mathbf{v}), \lambda_Y(\mathbf{v}))\}_{\mathbf{v} \in \mathbb{R}^d_{\text{unit}}}$. In order to illustrate the quantities defined above, we provide a toy example in Figure 5 by randomly generating the matrices C_X and C_Y. In Figure 5, the teal region forms the set $\mathcal{U}$, and the brown curve corresponds to elements in $\mathcal{S}_\lambda$. Note that $\mathcal{S}_\lambda$ forms the lower-right boundary of $\mathcal{U}$, which can also be inferred from the above definition.

Now let us consider directions that are returned by cPCA. Without loss of generality, we will focus our attention on the top cPC selected by cPCA (for different values of α).[1] For any contrastive analysis method to be reasonable, one would naturally require that the directions it generates lie in $\mathcal{S}_{\mathbf{v}}$. We show that this is indeed the case for cPCA. Furthermore, we show that the set of top cPCs with different values of α is actually identical to $\mathcal{S}_{\mathbf{v}}$. In other words, cPCA recovers all contrastive directions, yielding its optimality. This is stated as below (with proof provided in Supplementary Note 4.1):

[1] This is because, after selecting the first k cPCs, the $(k+1)$-th cPC is obtained by maximizing $\mathbf{v}^T(C_X - \alpha C_Y)\mathbf{v}$ over the space orthogonal to the first k cPCs. By rotating the space such that the first k components correspond to the first k dimensions, and then truncating the first k dimensions, the problem of selecting the $(k+1)$-th cPC is reduced to the same problem as finding the top cPC but with dimensionality $k-d$.

Theorem 1. *Let $\mathcal{S}_{\mathbf{v}}^{cPCA}$ be the set of top cPC of cPCA and let $\mathcal{S}_{\lambda}^{cPCA}$ be the corresponding set of target-background variance pairs:*

$$\mathcal{S}_{\mathbf{v}}^{cPCA} \stackrel{def}{=} \{\mathbf{v} : \exists \alpha \geq 0 \ s.t. \ \mathbf{v} \in \operatorname*{argmax}_{\mathbf{v}' \in \mathbb{R}^d_{unit}} \lambda(\mathbf{v}') - \alpha\sigma(\mathbf{v}')\},$$
$$\mathcal{S}_{\lambda}^{cPCA} \stackrel{def}{=} \{(\sigma(\mathbf{v}), \lambda(\mathbf{v})) : \mathbf{v} \in \mathcal{S}_{\mathbf{v}}^{cPCA}\}.$$

For $\mathcal{S}_{\mathbf{v}}$, $\mathcal{S}_{\lambda}$ in Definition 2, we have

$$\mathcal{S}_{\mathbf{v}}^{cPCA} = \mathcal{S}_{\mathbf{v}}, \qquad \mathcal{S}_{\lambda}^{cPCA} = \mathcal{S}_{\lambda}.$$

Remark 1. *(A geometric interpretation of α)* For the direction $\mathbf{v}$ selected by cPCA with the contrast parameter set to α, its variance pair $(\lambda_X(\mathbf{v}), \lambda_Y(\mathbf{v}))$ corresponds to the point of tangency of $\mathcal{S}_{\lambda}$ with a line of slope $1/\alpha$. For example, the left blue triangle in Figure 5 corresponds to the cPCA direction with $\alpha = 0.92$, and it is the point of tangency of the red curve $\mathcal{V}(\mathcal{S})$ and the blue line with slope $1.08(= 1/0.92)$. As a result, by varying α from zero to infinity, cPCA selects directions with variance pairs traveling from the lower-left end to the upper-right end of $\mathcal{S}_{\lambda}$.

This interpretation can be derived from the following observation. Consider any sequence $\alpha_n \downarrow \alpha$. Then there exists a sequence $\mathbf{v}_n$ such that $\mathbf{v}_n$ is the solution to (1) with alpha value α_n, and $\lambda_X(\mathbf{v}_n) \uparrow \lambda_X(\mathbf{v})$, $\lambda_Y(\mathbf{v}_n) \uparrow \lambda_Y(\mathbf{v})$. By Lemma 2,

$$\frac{1}{\alpha_n} \leq \frac{\lambda_Y(\mathbf{v}_n) - \lambda_Y(\mathbf{v})}{\lambda_X(\mathbf{v}_n) - \lambda_X(\mathbf{v})} \leq \frac{1}{\alpha},$$

giving

$$\lim_{n\to\infty} \frac{\lambda_Y(\mathbf{v}_n) - \lambda_Y(\mathbf{v})}{\lambda_X(\mathbf{v}_n) - \lambda_X(\mathbf{v})} = \frac{1}{\alpha}.$$

This implies that $(\lambda_X(\mathbf{v}), \lambda_Y(\mathbf{v}))$ is the point of tangency of $\mathcal{S}_{\lambda}$ and the slope-$\frac{1}{\alpha}$ tangent line.

Example 1. *(Simultaneously diagonalizable matrices)* A closed form representation of $\mathcal{S}_{\lambda}$ can be derived for the special case where the matrices C_X and C_Y are simultaneously diagonalizable. We derive it here to provide some intuition for the topology of the target-background variance pairs.

Let Q be the unitary matrix that diagonalize C_X and C_Y, i.e.

$$C_X = Q\Lambda_X Q^T, \qquad C_Y = Q\Lambda_Y Q^T,$$

where $\Lambda_X = \text{diag}(\lambda_{X,1}, \cdots, \lambda_{X,d})$, $\Lambda_Y = \text{diag}(\lambda_{Y,1}, \cdots, \lambda_{Y,d})$. Let $\mathbf{q}_1, \cdots, \mathbf{q}_d$ be the eigenvectors. Any unit vector can be written as $\mathbf{v} = \sum_i \sqrt{c_i}\mathbf{q}_i$, for $c_1, \cdots, c_d \geq 0$, $\sum_i c_i = 1$. Then the target and the background variances can be written as

$$\lambda_X(\mathbf{v}) = \mathbf{v}^T C_X \mathbf{v} = \sum_i c_i \lambda_{X,i},$$
$$\lambda_Y(\mathbf{v}) = \mathbf{v}^T C_Y \mathbf{v} = \sum_i c_i \lambda_{Y,i}.$$

Since the variance pair $(\lambda_X(\mathbf{v}), \lambda_Y(\mathbf{v}))$ is a convex combination of the variance pairs of eigenvectors $\{(\lambda_{X,i}, \lambda_{Y,i})\}_{i=1}^d$, the set of variance pairs $\{(\lambda_X(\mathbf{v}), \lambda_Y(\mathbf{v}))\}_{\mathbf{v}\in\mathbb{R}^d_{\text{unit}}}$ is the convex hull of $\{(\lambda_{X,i}, \lambda_{Y,i})\}_{i=1}^d$. Also $\mathcal{S}_\lambda$ is the lower-right boundary of the convex hull of $\{(\lambda_{X,i}, \lambda_{Y,i})\}_{i=1}^d$. We visualize this in Supplementary Figure 7 using randomly generated the simultaneously diagonalizable matrices C_X and C_Y.

As a result, $\mathcal{S}_\mathbf{v}$ can be written as follows. Let $\mathbf{q}_{(1)}, \cdots, \mathbf{q}_{(k)} \in \{\mathbf{q}_i\}_{i=1}^d$ be the eigenvectors whose variance pairs $(\lambda_{X,(j)}, \lambda_{Y,(j)})$ lie on the lower-right boundary of the convex hull of $\{(\lambda_{X,i}, \lambda_{Y,i})\}_{i=1}^d$, indexed in the ascending order of $\lambda_{X,(j)}$. Then

$$\mathcal{S}_\mathbf{v} = \{\mathbf{v} : \mathbf{v} = \sqrt{c}\mathbf{q}_{(j)} + \sqrt{1-c}\mathbf{q}_{(j+1)},\ for\ 0 \le c \le 1, 1 \le j \le k-1\}.$$

This implies that $\mathcal{S}_\mathbf{v}$ is a union of $(k-1)$ curved line segments of the form $\sqrt{c}\mathbf{q}_{(j)} + \sqrt{1-c}\mathbf{q}_{(j+1)}$, which is itself a curved line segment in the k dimensional subspace spanned by $\mathbf{q}_{(1)}, \cdots, \mathbf{q}_{(k)}$.

2.2 Convergence rate of the sample cPC

So far, the analysis concerns only the population cPC calculated based on the population covariance matrix $C = C_X - \alpha C_X$. In practice, we only have finite number of samples, say n data points from the target data and m data points from the background data. Let $\hat{C}_X$ and $\hat{C}_Y$ be the sample covariance matrices and let $\hat{C} = \hat{C}_X - \alpha\hat{C}_Y$. Then the sample cPC's are eigenvectors of $\hat{C}$. Here we characterize the convergence rate of the sample cPC to the population cPC.

Since the sample cPC corresponds to the eigenvector of the sample covariance matrix $\hat{C}$, the convergence property of the sample cPC is the same as that of the sample eigenvectors for a covariance matrix, which is well studied in previous literature[13, 14, 15]. In short, under mild assumptions, the sample cPC will converge to the population cPC when the sample size is larger than the dimensionality. We state this formally in the following theorem (with proof provided in Supplementary Note 2):

Theorem 2. *(Convergence rate of the sample cPC) Let $\hat{\mathbf{v}}^*$ be the first sample cPC and $\mathbf{v}^*$ be the first population cPC* [2]*. Assume that the entries of the target and the background data are sub-Gaussian with some fixed parameter, and the gap between the first and the second eigenvalue of C are bounded away from* 0*. Then,*

$$1 - |(\hat{\mathbf{v}}^*)^T\mathbf{v}^*| = O_p(\sqrt{\frac{d}{\min(n,m)}}),$$

where O_p denotes that the equation holds with high probability.

[2] Similar results can be shown for other cPC's by assuming an eigenvalue gap and using Wedin's Theorem.

2.3 A probabilistic interpretation

Suppose the target and the background data follow a Gaussian distribution. Then they can be written as linear combinations of standard Gaussian vectors $Z_i, U_i, Z_{i'}, V_{i'} \overset{i.i.d.}{\sim} \mathcal{N}(0, I)$, as well as noise vectors $\epsilon_i, \epsilon_{i'} \overset{i.i.d.}{\sim} \mathcal{N}(0, \sigma^2 I)$. The linear subspace can be determined as follows: Let $W_Y \in \mathbb{R}^{d \times p_Y}$ be the subspace unique to the background data, $W \in \mathbb{R}^{d \times p}$ be the rest of the subspace of the background data, and $W_X \in \mathbb{R}^{d \times p_X}$ be such that $W \bigcup W_X$ span the subspace of the target data. Then one can write

$$\begin{aligned} \mathbf{x}_i &= W Z_i + W_X U_i + \epsilon_i \\ \mathbf{y}_{i'} &= W Z_{i'} + W_Y V_{i'} + \epsilon_{i'}, \end{aligned}$$

where we note that $\text{span}(W \bigcup W_X) \bigcap \text{span}(W_Y) = \emptyset$.

Let $W_{X,\perp}$ be the subspace of W_X that is perpendicular to the subspace $\text{span}(W)$ and let $W_{X,\parallel}$ be that parallel to $\text{span}(W)$. With some technical derivation detailed in Supplementary Note 4.4, one can reach that

$$\mathbf{v}^* = \underset{\mathbf{v}}{\text{argmax}}\, \mathbf{v}^T \left(W_{X,\perp} W_{X,\perp}^T + W_{X,\parallel} W_{X,\parallel}^T + (1-\alpha) W W^T \right) \mathbf{v}. \tag{1}$$

Now (1) is readily interpretable. When α is small, $\mathbf{v}^*$ represents a trade-off between the space unique to the target data $\text{span}(W_{X,\perp})$ and the space shared between the two datasets $\text{span}(W)$. After α reach a threshold, $\mathbf{v}^*$ becomes the first eigenvector of $W_{X,\perp} W_{X,\perp}^T$, i.e. the first principal component of the space unique to the target data. Spefically, in the special case when $\text{span}(W_X)$ is orthogonal to $\text{span}(W)$, this threshold is 1. In other words, when $\alpha \geq 1$, $\mathbf{v}^*$ remains the first PC of the space unique to the target data, $\text{span}(W_{X,\perp})$.

3 Supplementary Note 3: Extension: Kernel cPCA

We extend cPCA to kernel cPCA, following the analogous extension of PCA to kernel PCA[16]. Full details are in Supplementary Note 4.5.

Consider the nonlinear transformation $\Phi : \mathbb{R}^d \mapsto F$ that maps the data to some feature space F. We assume that the mapped data, $\Phi(\mathbf{x}_1), \cdots, \Phi(\mathbf{x}_n)$, $\Phi(\mathbf{y}_1), \cdots, \Phi(\mathbf{y}_m)$, is centered, i.e. $\sum_{i=1}^n \Phi(\mathbf{x}_i) = \sum_{j=1}^m \Phi(\mathbf{y}_j) = 0$. (The general case is considered in Supplementary Note 4.5.)

The covariance matrices for the target and the background can be written as

$$\bar{C}_X = \frac{1}{n}\sum_{i=1}^{n} \Phi(\mathbf{x}_i)\Phi(\mathbf{x}_i)^T, \qquad \bar{C}_Y = \frac{1}{m}\sum_{j=1}^{m} \Phi(\mathbf{y}_j)\Phi(\mathbf{y}_j)^T.$$

cPCA on the transformed data solves for the eigenvectors of $(\bar{C}_X - \alpha\bar{C}_Y)\mathbf{v}$, where the k-th eigenvector is the k-th cPC, but this is inefficient if the dimensionality of F is large.

We next describe the kernel cPCA algorithm, which allows us to efficiently perform contrastive analysis on the transformed data.

Let $N = n+m$ and denote the data as $(\mathbf{z}_1, \cdots, \mathbf{z}_N) = (\mathbf{x}_1, \cdots, \mathbf{x}_n, \mathbf{y}_1, \cdots, \mathbf{y}_m)$. Define the kernel matrix K to have the ij-th element $K_{ij} = \Phi(\mathbf{z}_i)\cdot\Phi(\mathbf{z}_j)$, and write it in form of a block matrix as

$$K = \begin{bmatrix} K_X & K_{XY} \\ K_{YX} & K_Y \end{bmatrix}, \tag{2}$$

where $K_X \in \mathbb{R}^{n\times n}$, $K_Y \in \mathbb{R}^{m\times m}$ are the sub-kernels corresponding to $\mathbf{x}_1, \cdots, \mathbf{x}_n$, and $\mathbf{y}_1, \cdots, \mathbf{y}_m$, respectively.

As derived in Supplementary Note 4.5, instead of directly calculating the eigenvectors of $(\bar{C}_X - \alpha\bar{C}_Y)\mathbf{v}$, we can consider its dual representation $\mathbf{v} = \sum_{i=1}^N a_i\Phi(\mathbf{z}_i)$, and solve a_i's via the following eigenvalue problem for non-zero eigenvalues:

$$\lambda\mathbf{a} = \tilde{K}\mathbf{a}, \tag{3}$$

where the first eigenvector $\mathbf{a}^{(1)}$ corresponds to the first cPC, and

$$\tilde{K} = \begin{bmatrix} \frac{1}{n}K_X & \frac{1}{n}K_{XY} \\ -\frac{\alpha}{m}K_{YX} & -\frac{\alpha}{m}K_Y \end{bmatrix}.$$

To make $\|\mathbf{v}\| = 1$, we require $\mathbf{a}^T K\mathbf{a} = 1$. Finally, we can project the data onto the k-th cPC by

$$[\mathbf{v}^{(k)}\cdot\Phi(\mathbf{z}_1), \cdots, \mathbf{v}^{(k)}\cdot\Phi(\mathbf{z}_N)] = K\mathbf{a}^{(k)}.$$

Note that in the above calculation, the kernel can be constructed via some kernel function $h(\cdot,\cdot)$ as $K_{ij} = h(\mathbf{z}_i, \mathbf{z}_j)$, and the projected data can be computed as $K\mathbf{a}^{(k)}$. As a result, by kernel cPCA, we can actually perform cPCA in the feature space without explicitly computing the non-linearly transformed data.

Example 2. *(A toy example)* In this dataset, $d = 10$, and the first two dimensions x_1, x_2 contain the subgroup structure in the target data. As shown in Supplementary Figure 8a, the two subgroups can not be linearly separated directly. However, Supplementary Figure 8b shows that they can be linearly separated if we project the data on the non-linear features $\phi(x_1) = x_1^2$ and $\phi(x_2) = x_2^2$.

We tested PCA, cPCA, kernel PCA, kernel cPCA, using the polynomial kernel $K(\mathbf{x}, \mathbf{y}) = (\mathbf{x}^T \mathbf{y} + 1)^2$ for the latter two to address the non-linear mapping. As shown in Supplementary Figure 8c-f, both cPCA and kernel cPCA recover the subspace that contains the subgroup structure, but only kernel cPCA produces a subspace where the two subgroups are linearly separable.

Remark 2. It is often challenging to get kernel cPCA work effectively in practice. This is because kernel cPCA is implicitly performing cPCA in the transformed feature space. However, the kernel generally induces a feature space with many correlated features, creating a large null space in the background data. Since cPCA does not have a penalty for directions in this null space and this null space is large, the background dataset will not be very effective at canceling out directions in the target. We plan to address this issue in the future work.

4 Supplementary Note 4: Technical Proofs

4.1 Proof of Theorem 1

Proof. Since $\mathcal{S}_\lambda$ and $\mathcal{S}_\lambda^{cPCA}$ are continuous images of $\mathcal{S}_\mathbf{v}$ and $\mathcal{S}_\mathbf{v}^{cPCA}$, it suffices to just show $\mathcal{S}_\mathbf{v}^{cPCA} = \mathcal{S}_\mathbf{v}$.

We first show that $\mathcal{S}_\mathbf{v}^{cPCA} \subset \mathcal{S}_\mathbf{v}$. Consider any $\mathbf{v} \in \mathcal{S}_\mathbf{v}^{cPCA}$ that is the solution of Equation (1) in the main article with alpha value α. For any $\mathbf{u} \in \mathbb{R}^d_{\text{unit}}$, we have

$$\mathbf{v}^T(C_X - \alpha C_Y)\mathbf{v} \geq \mathbf{u}^T(C_X - \alpha C_Y)\mathbf{u}, \tag{4}$$

which can be rewritten as

$$\lambda_X(\mathbf{v}) - \lambda_X(\mathbf{u}) \geq \alpha(\lambda_Y(\mathbf{v}) - \lambda_Y(\mathbf{u})). \tag{5}$$

Then there are three possibilities of the relations between the variance pairs of $\mathbf{v}$ and $\mathbf{u}$:

1. $\lambda_X(\mathbf{v}) > \lambda_X(\mathbf{u})$,
2. $\lambda_X(\mathbf{v}) = \lambda_X(\mathbf{u}), \quad \lambda_Y(\mathbf{v}) \leq \lambda_Y(\mathbf{u})$,
3. $\lambda_X(\mathbf{v}) < \lambda_X(\mathbf{u}), \quad \lambda_Y(\mathbf{v}) < \lambda_Y(\mathbf{u})$.

In all three cases, $\mathbf{u}$ can not be more contrastive than $\mathbf{v}$. Thus $\mathbf{v} \in \mathcal{S}_\mathbf{v}$ and we can conclude that $\mathcal{S}_\mathbf{v}^{cPCA} \subset \mathcal{S}_\mathbf{v}$.

Next we show $\mathcal{S}_\mathbf{v} \subset \mathcal{S}_\mathbf{v}^{cPCA}$ by contradiction. Suppose there exists $\mathbf{v} \in \mathcal{S}_\mathbf{v}$ such that $\mathbf{v} \notin \mathcal{S}_\mathbf{v}^{cPCA}$. Let us define

$$\begin{aligned} \mathbf{v}_l &\in \operatorname*{argmax}_{\mathbf{u}:\ \mathbf{u} \in \mathcal{S}_\mathbf{v}^{cPCA}, \lambda_X(\mathbf{u}) < \lambda_X(\mathbf{v})} \lambda_X(\mathbf{u}) \\ \mathbf{v}_u &\in \operatorname*{argmin}_{\mathbf{u}:\ \mathbf{u} \in \mathcal{S}_\mathbf{v}^{cPCA}, \lambda_X(\mathbf{u}) > \lambda_X(\mathbf{v})} \lambda_X(\mathbf{u}). \end{aligned} \tag{6}$$

The existence of $\mathbf{v}_l$ can be argued by showing the set $\{\mathbf{u} : \ \mathbf{u} \in \mathcal{S}_\mathbf{v}^{cPCA}, \lambda_X(\mathbf{u}) < \lambda_X(\mathbf{v})\}$ is both nonempty and compact. The nonemptiness is because $\lambda_X(\mathbf{v}) > \min_{\mathbf{u} \in \mathcal{S}_\mathbf{v}^{cPCA}} \lambda_X(\mathbf{u})$, which can be seen be contradiction. The compactness is because both $\mathcal{S}_\mathbf{v}^{cPCA}$ and $\mathcal{S}_\lambda^{cPCA}$ are compact by Lemma 1, and $\mathbf{v} \notin \mathcal{S}_\mathbf{v}^{cPCA}$. The existence of $\mathbf{v}_u$ can be shown in a similar fashion.

Furthermore, let $\alpha' = \frac{\lambda_X(\mathbf{v}_u) - \lambda_X(\mathbf{v}_l)}{\lambda_Y(\mathbf{v}_l) - \lambda_Y(\mathbf{v}_l)}$. We next show that both $\mathbf{v}_l$ and $\mathbf{v}_u$ are solutions to Equation (1) (main article) with alpha value α'.

Since $\mathbf{v}_l$, $\mathbf{v}_u \in \mathcal{S}_\mathbf{v}^{cPCA}$, as shown previously, $\mathbf{v}_l$, $\mathbf{v}_u \in \mathcal{S}_\mathbf{v}$. Then according to Lemma 2,

$$\sup_{\substack{\mathbf{u}:\ \mathbf{u} \in \mathcal{S}_\mathbf{v}, \\ \lambda_X(\mathbf{u}) < \lambda_X(\mathbf{v}_l)}} \frac{\lambda_Y(\mathbf{v}_l) - \lambda_Y(\mathbf{u})}{\lambda_X(\mathbf{v}_l) - \lambda_X(\mathbf{u})} \leq \inf_{\substack{\mathbf{u}:\ \mathbf{u} \in \mathcal{S}_\mathbf{v}, \\ \lambda_X(\mathbf{u}) > \lambda_X(\mathbf{v}_l)}} \frac{\lambda_Y(\mathbf{v}_l) - \lambda_Y(\mathbf{u})}{\lambda_X(\mathbf{v}_l) - \lambda_X(\mathbf{u})}$$

$$\sup_{\substack{\mathbf{u}:\ \mathbf{u} \in \mathcal{S}_\mathbf{v}, \\ \lambda_X(\mathbf{u}) < \lambda_X(\mathbf{v}_u)}} \frac{\lambda_Y(\mathbf{v}_u) - \lambda_Y(\mathbf{u})}{\lambda_X(\mathbf{v}_u) - \lambda_X(\mathbf{u})} \leq \inf_{\substack{\mathbf{u}:\ \mathbf{u} \in \mathcal{S}_\mathbf{v}, \\ \lambda_X(\mathbf{u}) > \lambda)_X(\mathbf{v}_u)}} \frac{\lambda_Y(\mathbf{v}_u) - \lambda_Y(\mathbf{u})}{\lambda_X(\mathbf{v}_u) - \lambda_X(\mathbf{u})}$$

Then $\mathbf{v}_u$ is inside the inf term in the first equation above, and $\mathbf{v}_l$ is inside the sup term in the second equation above, both of which have the corresponding ratio $1/\alpha'$. Then,

$$\sup_{\substack{\mathbf{u}:\ \mathbf{u}\in\mathcal{S}_{\mathbf{v}},\\ \lambda_X(\mathbf{u})<\lambda_X(\mathbf{v}_l)}} \frac{\lambda_Y(\mathbf{v}_l)-\lambda_Y(\mathbf{u})}{\lambda_X(\mathbf{v}_l)-\lambda_X(\mathbf{u})} \leq \frac{1}{\alpha'} \leq \inf_{\substack{\mathbf{u}:\ \mathbf{u}\in\mathcal{S}_{\mathbf{v}},\\ \lambda_X(\mathbf{u})>\lambda_X(\mathbf{v}_u)}} \frac{\lambda_Y(\mathbf{v}_u)-\lambda_Y(\mathbf{u})}{\lambda_X(\mathbf{v}_u)-\lambda_X(\mathbf{u})} \tag{7}$$

To show that $\mathbf{v}_l$ and $\mathbf{v}_u$ are solutions to Equation (1) (main article) with alpha value α', it suffices to show that $\forall \mathbf{u} \in \mathcal{S}_{\mathbf{v}}$,

$$\begin{aligned} \mathbf{v}_l^T(C_X-\alpha' C_Y)\mathbf{v}_l' &\geq \mathbf{u}^T(C_X-\alpha' C_Y)\mathbf{u}' \\ \mathbf{v}_u^T(C_X-\alpha' C_Y)\mathbf{v}_u' &\geq \mathbf{u}^T(C_X-\alpha' C_Y)\mathbf{u}'. \end{aligned}$$

We consider three cases of $\mathbf{u}$. For any $\mathbf{u} \in \mathcal{S}_{\mathbf{v}}$ such that $\lambda_X(\mathbf{u}) < \lambda_X(\mathbf{v}_l)$, we also know $\lambda_Y(\mathbf{u}) < \lambda_Y(\mathbf{v}_l)$. According to (7),

$$\frac{\lambda_Y(\mathbf{v}_l)-\lambda_Y(\mathbf{u})}{\lambda_X(\mathbf{v}_l)-\lambda_X(\mathbf{u})} \leq \frac{1}{\alpha'},$$

which is equivalent to

$$\mathbf{v}_l^T(C_X-\alpha' C_Y)\mathbf{v}_l' \geq \mathbf{u}^T(C_X-\alpha' C_Y)\mathbf{u}'.$$

Moreover, since $\frac{1}{\alpha'} = \frac{\lambda_Y(\mathbf{v}_u)-\lambda_Y(\mathbf{v}_l)}{\lambda_X(\mathbf{v}_u)-\lambda_X(\mathbf{v}_l)}$, we also have that

$$\frac{\lambda_Y(\mathbf{v}_u)-\lambda_Y(\mathbf{u})}{\lambda_X(\mathbf{v}_u)-\lambda_X(\mathbf{u})} \leq \frac{1}{\alpha'},$$

giving that

$$\mathbf{v}_u^T(C_X-\alpha' C_Y)\mathbf{v}_u' \geq \mathbf{u}^T(C_X-\alpha' C_Y)\mathbf{u}'.$$

Second, the same reasoning can be applied to the case of $\mathbf{u} \in \mathcal{S}_{\mathbf{v}}$ such that $\lambda_X(\mathbf{u}) > \lambda_X(\mathbf{v}_u)$

Third, for any $\mathbf{u} \in \mathcal{S}_{\mathbf{v}}$ such that $\lambda_X(\mathbf{v}_l) < \lambda_X(\mathbf{u}) < \lambda_X(\mathbf{v}_u)$, by definition (7), $\mathbf{u} \notin \mathcal{S}_{\mathbf{v}}^{cPCA}$, and hence can not be the solution to Equation (1) (main article) with alpha value α'. Therefore, $\mathbf{v}_l$ and $\mathbf{v}_u$ are solutions to Equation (1) (main article) with alpha value α'.

Then both $\mathbf{v}_l$ and $\mathbf{v}_u$ are eigenvectors of $C_X - \alpha' C_Y$ with the same eigenvalue. Then there exists $\mathbf{v}'$ in this eigenspace such that $\lambda_X(\mathbf{v}_l) < \lambda_X(\mathbf{v}') < \lambda_X(\mathbf{v}_u)$. We note that it is also the solution to Equation (1) in the main article with alpha value α' and is hence in $\mathcal{S}_{\mathbf{v}}^{cPCA}$. This contradicts the definition (6), which completes the proof. □

4.2 Ancillary Lemmas for Theorem 1

Lemma 1. *$\mathcal{S}_{\mathbf{v}}^{cPCA}$ and $\mathcal{S}_{\lambda}^{cPCA}$ are compact.*

Proof. (Proof of Lemma 1) Consider any sequence of directions $\mathbf{v}_n$ in $\mathcal{S}_{\mathbf{v}}^{cPCA}$ that converges to $\mathbf{v}$. There exists a corresponding sequence of alpha's α_n with limit α, where $\mathbf{v}_n$ is the solution of Equation (1) in the main article with α_n. Then

$$\begin{aligned}\mathbf{v}^T(C_X - \alpha C_Y)\mathbf{v} &= \lim_{n\to\infty} \mathbf{v}_n^T(C_X - \alpha_n C_Y)\mathbf{v}_n \\ &= \lim_{n\to\infty} \max_{\mathbf{u}\in\mathbb{R}^d_{\text{unit}}} \mathbf{u}^T(C_X - \alpha_n C_Y)\mathbf{u} \\ &= \max_{\mathbf{u}\in\mathbb{R}^d_{\text{unit}}} \mathbf{u}^T(C_X - \alpha C_Y)\mathbf{u},\end{aligned}$$

giving that $\mathbf{v} \in \mathcal{S}_{\mathbf{v}}^{cPCA}$. Hence $\mathcal{S}_{\mathbf{v}}^{cPCA}$ is compact. Finally, being the continuous image of a compact set, $\mathcal{S}_{\lambda}^{cPCA}$ is also compact. □

Lemma 2. *If $\mathbf{v} \in \mathcal{S}_{\mathbf{v}}$ and $\mathbf{v}$ is the solution to Equation (1) in the main article with value α, then*

$$\sup_{\substack{\mathbf{u}:\ \mathbf{u}\in\mathcal{S}_{\mathbf{v}},\\ \lambda_X(\mathbf{u})<\lambda_X(\mathbf{v})}} \frac{\lambda_Y(\mathbf{v}) - \lambda_Y(\mathbf{u})}{\lambda_X(\mathbf{v}) - \lambda_X(\mathbf{u})} \le \frac{1}{\alpha} \le \inf_{\substack{\mathbf{u}:\ \mathbf{u}\in\mathcal{S}_{\mathbf{v}},\\ \lambda_X(\mathbf{u})>\lambda_X(\mathbf{v})}} \frac{\lambda_Y(\mathbf{v}) - \lambda_Y(\mathbf{u})}{\lambda_X(\mathbf{v}) - \lambda_X(\mathbf{u})}. \tag{8}$$

Proof. (Proof of Lemma 2) For any $\mathbf{u} \in \mathcal{S}_{\mathbf{v}}$, we have

$$\mathbf{v}^T(C_X - \alpha C_Y)\mathbf{v} \ge \mathbf{u}^T(C_X - \alpha C_Y)\mathbf{u},$$

which is equivalent to

$$\lambda_X(\mathbf{v}) - \lambda_X(\mathbf{u}) \ge \alpha(\lambda_Y(\mathbf{v}) - \lambda_Y(\mathbf{u})). \tag{9}$$

Since $\mathbf{v}, \mathbf{u} \in \mathcal{S}_{\mathbf{v}}$, $\lambda_X(\mathbf{v}) > \lambda_X(\mathbf{u})$ implies $\lambda_Y(\mathbf{v}) > \lambda_Y(\mathbf{u})$ and vice versa. As (9) holds for all $\mathbf{u} \in \mathcal{S}_{\mathbf{v}}$, this gives (8). □

4.3 Proof of Theorem 2

Proof. According to the standard results for covariance matrix concentration, e.g. Corollary 5.50 in [13], with probability at least $1 - 4e^{-d}$ (we refer to this as with high probability),

$$\begin{aligned}\|\hat{C}_X - C_X\|_{op} &\le O(\sqrt{\frac{d}{n}}) \le O(\sqrt{\frac{d}{\min(n,m)}}) \\ \|\hat{C}_Y - C_Y\|_{op} &\le O(\sqrt{\frac{d}{m}}) \le O(\sqrt{\frac{d}{\min(n,m)}}),\end{aligned}$$

where $\|\cdot\|_{op}$ denotes the matrix operation norm. Furthermore, we have that

$$\begin{aligned}\|\hat{C} - C\|_{op} &= \|(\hat{C}_X - \alpha\hat{C}_Y) - (C_X - \alpha C_Y)\|_{op} \\ &\le \|\hat{C}_X - C_X\|_{op} + \alpha\|\hat{C}_Y - C_Y\|_{op} = O(\sqrt{\frac{d}{\min(n,m)}}).\end{aligned}$$

Finally, by Wedin's theorem,

$$1 - |(\hat{\mathbf{v}}^*)^T \mathbf{v}^*| \leq \frac{\|\hat{C} - C\|_{op}}{\lambda_1(C) - \lambda_2(X) - \|\hat{C} - C\|_{op}} = O(\sqrt{\frac{d}{\min(n,m)}}),$$

where $\lambda_1(C)$ and $\lambda_2(C)$ are the first and the second eigenvalues of C and we assume $\lambda_1(C) - \lambda_2(X)$ is a constant bounded away from 0. □

4.4 Proof regarding Supplementary Note 2.3

Proof. Next, the covariance matrices can be written as

$$C_X = WW^T + W_X W_X^T + \sigma^2 I, \quad C_Y = WW^T + W_Y W_Y^T + \sigma^2 I.$$

Furthermore,

$$C_X - \alpha C_Y = W_X W_X^T - \alpha W_Y W_Y^T + (1-\alpha) WW^T + (1-\alpha)\sigma^2 I.$$

Now let us consider the first cPC $\mathbf{v}^*$. One can write

$$\begin{aligned}
\mathbf{v}^* &= \underset{\mathbf{v}}{\operatorname{argmax}}\, \mathbf{v}^T \left(C_X - \alpha C_Y\right) \mathbf{v} \\
&= \underset{\mathbf{v}}{\operatorname{argmax}}\, \mathbf{v}^T \left(W_X W_X^T - \alpha W_Y W_Y^T + (1-\alpha) WW^T + (1-\alpha)\sigma^2 I\right) \mathbf{v} \\
&= \underset{\mathbf{v}}{\operatorname{argmax}}\, \mathbf{v}^T \left(W_X W_X^T - \alpha W_Y W_Y^T + (1-\alpha) WW^T\right) \mathbf{v} \\
&= \underset{\mathbf{v}}{\operatorname{argmax}}\, \mathbf{v}^T \left(W_X W_X^T + (1-\alpha) WW^T\right) \mathbf{v},
\end{aligned}$$

where the third equality is because $\mathbf{v}^T(\sigma^2 I)\mathbf{v}$ is homogeneous across all directions, and the forth equality is because the solution only lie in the null space of W_Y. Furthermore write $W_X = W_{X,\parallel} + W_{X,\perp}$, for $W_{X,\parallel}$ parallel and $W_{X,\perp}$ perpendicular to the subspace spanned by W, we reach that the first cPC

$$\mathbf{v}^* = \underset{\mathbf{v}}{\operatorname{argmax}}\, \mathbf{v}^T \left(W_{X,\perp} W_{X,\perp}^T + W_{X,\parallel} W_{X,\parallel}^T + (1-\alpha) WW^T\right) \mathbf{v}. \tag{10}$$

□

4.5 Derivation of Kernel cPCA

Assume for the moment that the mapped data, $\Phi(\mathbf{x}_1), \cdots, \Phi(\mathbf{x}_n)$, $\Phi(\mathbf{y}_1), \cdots, \Phi(\mathbf{y}_m)$, is centered i.e., $\sum_{i=1}^n \Phi(\mathbf{x}_i) = \sum_{j=1}^m \Phi(\mathbf{y}_j) = 0$. The non-centered case will be considered in the end. The covariance matrices for the target data and background data are

$$\bar{A} = \frac{1}{n} \sum_{i=1}^n \Phi(\mathbf{x}_i)\Phi(\mathbf{x}_i)^T, \quad \bar{B} = \frac{1}{m} \sum_{j=1}^m \Phi(\mathbf{y}_j)\Phi(\mathbf{y}_j)^T.$$

The contrastive components should satisfy

$$\lambda \mathbf{v} = (\bar{A} - \alpha \bar{B})\mathbf{v}, \tag{11}$$

where the k-th eigenvector corresponds to the k-th contrastive principal component. Let $N = n + m$ and define the data $\mathbf{z}_1, \cdots, \mathbf{z}_N$ as

$$\mathbf{z}_l = \begin{cases} \mathbf{x}_l, & if\ 1 \le l \le n \\ \mathbf{y}_{l-n} & otherwise \end{cases}.$$

As all contrastive principal components $\mathbf{v}$ lie in the span of $\Phi(\mathbf{z}_1, \cdots, \mathbf{z}_N)$, there exists $\mathbf{a} = (a_1, \cdots, a_l) \in \mathbb{R}^N$ such that $\mathbf{v}$ can be written as

$$\mathbf{v} = \sum_{k=1}^{N} a_k \Phi(\mathbf{z}_k). \tag{12}$$

Also, instead of (11), we can consider the equivalent system

$$\lambda \Phi(\mathbf{z}_l) \cdot \mathbf{v} = \Phi(\mathbf{z}_l) \cdot (\bar{A} - \alpha \bar{B})\mathbf{v}, \quad l = 1, \cdots, N. \tag{13}$$

Substituting (12) into (13), we have

$$\lambda \Phi(\mathbf{z}_l) \cdot \sum_{k=1}^{N} a_k \Phi(\mathbf{z}_k) = \Phi(\mathbf{z}_l) \cdot (\bar{A} - \alpha \bar{B}) \sum_{k=1}^{N} a_k \Phi(\mathbf{z}_k), \quad for\ l = 1, \cdots, N. \tag{14}$$

Define the $N \times N$ kernel matrix K by

$$K_{ij} = \Phi(\mathbf{z}_i) \cdot \Phi(\mathbf{z}_j), \tag{15}$$

and further define the $N \times N$ matrices K^A, K^B by

$$K_{ij}^A = \begin{cases} K_{ij}, & if\ 1 \le i \le n \\ 0 & otherwise \end{cases},$$

$$K_{ij}^B = \begin{cases} 0, & if\ 1 \le i \le n \\ K_{ij} & otherwise \end{cases}$$

Stacking all N equations together, the LHS of (14) is equal to $\lambda K\mathbf{a}$. It is also not hard to verify the RLS is equal to $K(\frac{1}{n}K^A - \frac{\alpha}{m}K^B)\mathbf{a}$. The we can rewrite the linear system (14) as

$$\lambda K\mathbf{a} = K(\frac{1}{n}K^A - \frac{\alpha}{m}K^B)\mathbf{a}. \tag{16}$$

To find the solution of (16), we solve the eigenvalue problem

$$\lambda \mathbf{a} = (\frac{1}{n}K^A - \frac{\alpha}{m}K^B)\mathbf{a} \tag{17}$$

for non-zero eigenvalues. Clearly all solutions of (17) do satisfy (16). Also, the solutions of (17) and those of (16) differ up to a term lying in the null space of K. Since the projection of the data on $\mathbf{v}$ is

$$[\Phi(\mathbf{z}_1) \cdot \mathbf{v}, \cdots, \Phi(\mathbf{z}_N) \cdot \mathbf{v}]^T = K\mathbf{a}, \tag{18}$$

any term lying in the null space of K does not affect the projected result. Hence to solve (16), we can equivalently solve (17). Finally, to impose the constraint that $\|\mathbf{v}\| = 1$, we equivalently require

$$\mathbf{a}^T K\mathbf{a} = 1. \tag{19}$$

Finally, as mentioned before, the projection of the data onto the q-th contrastive principal component can be written as $K\mathbf{a}^{(q)}$ as (18).

The centering assumption can be dropped as follows. Now assume that $\Phi(\mathbf{x}_i)$ and $\Phi(\mathbf{y}_j)$ has some general mean $\mu_X = \frac{1}{n}\sum_{i=1}^{n}\Phi(\mathbf{x}_i)$ and $\mu_Y = \frac{1}{m}\sum_{j=1}^{m}\Phi(\mathbf{y}_j)$. Let the non-centered kernel matrix K be the same as (15), and let it be partitioned into

$$K = \begin{bmatrix} K_X & K_{XY} \\ K_{YX} & K_Y \end{bmatrix}, \tag{20}$$

according to if the elements $\mathbf{z}_i$ and $\mathbf{z}_j$ belong to the target or the background data. Then the kernel matrix K can centered as

$$K_{center} = \begin{bmatrix} K_{X,center} & K_{XY,center} \\ K_{YX,center} & K_{Y,center} \end{bmatrix}, \tag{21}$$

where

$$\begin{aligned}
K_{X,center} &= K_X - \mathbf{1}_n K_X - K_X \mathbf{1}_n + \mathbf{1}_n K_X \mathbf{1}_n \\
K_{XY,center} &= K_{YX} - \mathbf{1}_m K_{YX} - K_{YX} \mathbf{1}_n + \mathbf{1}_m K_{YX} \mathbf{1}_n \\
K_{YX,center} &= K_{YX} - \mathbf{1}_m K_{YX} - K_{YX} \mathbf{1}_n + \mathbf{1}_m K_{YX} \mathbf{1}_n \\
K_{Y,center} &= K_Y - \mathbf{1}_m K_Y - K_Y \mathbf{1}_m + \mathbf{1}_m K_X \mathbf{1}_m,
\end{aligned}$$

and $\mathbf{1}_n$ and $\mathbf{1}_m$ has all elements $\frac{1}{n}$ and $\frac{1}{m}$ respectively.

Supplementary References

[1] Barshan, E., Ghodsi, A., Azimifar, Z. & Jahromi, M. Z. Supervised principal component analysis: Visualization, classification and regression on subspaces and submanifolds. *Pattern Recognition* **44**, 1357–1371 (2011).

[2] Izenman, A. J. Linear discriminant analysis. In *Modern multivariate statistical techniques*, 237–280 (Springer, 2013).

[3] Mika, S., Ratsch, G., Weston, J., Scholkopf, B. & Mullers, K.-R. Fisher discriminant analysis with kernels. In *Neural networks for signal processing IX, 1999. Proceedings of the 1999 IEEE signal processing society workshop.*, 41–48 (Ieee, 1999).

[4] Cox, M. A. & Cox, T. F. Multidimensional scaling. *Handbook of Data Visualization* 315–347 (2008).

[5] Zhou, Z., Li, X., Wright, J., Candes, E. & Ma, Y. Stable principal component pursuit. In *Information Theory Proceedings (ISIT), 2010 IEEE International Symposium on*, 1518–1522 (IEEE, 2010).

[6] Jolliffe, I. T. Principal component analysis and factor analysis. In *Principal component analysis*, 115–128 (Springer, 1986).

[7] Hyvärinen, A., Karhunen, J. & Oja, E. *Independent component analysis*, vol. 46 (John Wiley & Sons, 2004).

[8] Pedregosa, F. *et al.* Scikit-learn: Machine learning in Python. *Journal of Machine Learning Research* **12**, 2825–2830 (2011).

[9] Joliffe, I. & Morgan, B. Principal component analysis and exploratory factor analysis. *Statistical methods in medical research* **1**, 69–95 (1992).

[10] Wold, S., Esbensen, K. & Geladi, P. Principal component analysis. *Chemometrics and intelligent laboratory systems* **2**, 37–52 (1987).

[11] van den Berg, R. A., Hoefsloot, H. C., Westerhuis, J. A., Smilde, A. K. & van der Werf, M. J. *BMC Genomics* **7**, 142 (2006).

[12] Banos, O. *et al.* Design, implementation and validation of a novel open framework for agile development of mobile health applications. *BioMedical Engineering OnLine* **14**, S6 (2015).

[13] Vershynin, R. Introduction to the non-asymptotic analysis of random matrices. *arXiv preprint arXiv:1011.3027* (2010).

[14] Vershynin, R. How close is the sample covariance matrix to the actual covariance matrix? *Journal of Theoretical Probability* **25**, 655–686 (2012).

[15] Lee, S., Zou, F. & Wright, F. A. Convergence and prediction of principal component scores in high-dimensional settings. *Annals of statistics* **38**, 3605 (2010).

[16] Schölkopf, B., Smola, A. & Müller, K.-R. Kernel principal component analysis. In *International Conference on Artificial Neural Networks*, 583–588 (Springer, 1997).
